# Theories about Developmental Dyslexia

**DOI:** 10.3390/brainsci13020208

**Published:** 2023-01-26

**Authors:** John Stein

**Affiliations:** Department of Physiology, Anatomy & Genetics, Oxford University, Oxford OX1 3PT, UK; john.stein@dpag.ox.ac.uk

**Keywords:** dyslexia, reading, phonology, visual, auditory, temporal processing, magnocellular, parvocellular

## Abstract

Despite proving its usefulness for over a century, the concept of developmental dyslexia (DD) is currently in severe disarray because of the recent introduction of the phonological theory of its causation. Since mastering the phonological principle is essential for all reading, failure to do so cannot be used to distinguish DD from the many other causes of such failure. To overcome this problem, many new psychological, signal detection, and neurological theories have been introduced recently. All these new theories converge on the idea that DD is fundamentally caused by impaired signalling of the timing of the visual and auditory cues that are essential for reading. These are provided by large ‘magnocellular’ neurones which respond rapidly to sensory transients. The evidence for this conclusion is overwhelming. Especially convincing are intervention studies that have shown that improving magnocellular function improves dyslexic children’s reading, together with cohort studies that have demonstrated that the magnocellular timing deficit is present in infants who later become dyslexic, long before they begin learning to read. The converse of the magnocellular deficit in dyslexics may be that they gain parvocellular abundance. This may often impart the exceptional ‘holistic’ talents that have been ascribed to them and that society needs to nurture.

## 1. Introduction

The current definition of developmental dyslexia (DD) is highly unsatisfactory [1]. Even though it was accepted for over 100 years that some children have a genetically based impaired development of left hemisphere connections that normally mediate reading and spelling, yet their speech and oral comprehension develop normally, the introduction of the phonological theory of dyslexia means that it is now impossible for DD to be distinguished from other causes of failure to learn to read. Hence, some people now argue that it may not exist at all [2]. On the contrary, in this review of current theories about the causes of DD, I hope to convince the reader that the 19th Century. speculations about the existence of a developmental form of dyslexia have turned out to be absolutely correct and that most of the evidence accruing from modern advances in neuroscience supports this view.

The word ‘dyslexia’ was derived by Rudolph Berlin in 1884 [3] from the Greek ‘dys’ and ‘lexis’ (disordered words) to apply to patients in whom strokes had destroyed their ability to read and spell, yet left their speech and oral comprehension intact. We now call this ‘acquired’ dyslexia. A few years later, Hinshelwood [4] and Pringle Morgan [5] borrowed this concept to speculate that there might be a developmental form of dyslexia, which would explain why some children failed to develop the normal ‘connectome’ that mediates reading, despite developing normal speech and oral comprehension. They were well aware that this selective deficit implied that reading must employ at least some anatomically distinct pathways that are not used for speech and language comprehension. Recent functional imaging findings have confirmed their conjecture comprehensively [6]. They also recognised that developmental dyslexia was probably genetically based because of the strong family histories of reading difficulties in these children’s close relatives [7].

For over a century, developmental dyslexia was recognised by neurologists and other doctors as a useful diagnosis for which the key criteria were a significant discrepancy between a child’s backward reading, yet normal or high speech and oral comprehension and reasoning skills, together with a strong family history. However, since the turn of the millennium, this consensus has been thoroughly undermined.

## 2. Current Situation

First, responsibility for the diagnosis of DD transferred from medics to educational psychologists, because they became the first people to see potentially dyslexic children. In general, they were not trained in neuroscience and some became convinced that a diagnosis of DD failed to lead to any demonstrable improvement in teaching the recipients to learn to read. Thus, they began to react against the so called ‘medical model’ of reading difficulties and transferred their allegiance more to computational models that were often divorced from the neural machinery mediating them [8].

They were also highly influenced by the linguistic revolution introduced by Chomsky, in particular about the nature of phonemes [9]. To simplify, the essence of reading is to learn to translate letters into the sounds they stand for, which are ‘phonemes’. However, phonemes do not actually exist as a consistent, standard acoustic signal. Their acoustic form varies according to the preceding and succeeding sounds in the spoken word; thus, children have to learn a semi abstract concept, the ‘phonological principle’. Indeed, it turned out that children cannot learn it at all until they have learnt that the written forms of words consist visually of sequences of separate letters [10]. Only after this can they understand that the spoken form of a word can also be broken down into a sequence of sounds, phonemes, that the letters represent. Unlike speaking, this is not automatic; it is difficult, and most children have to be taught how it works. This new emphasis on phonology meant that DD became attributed entirely to failure to learn the phonological principle properly, and the ‘phonological theory’ of dyslexia became dominant [11].

Importantly, however, failure to master the phonological principle applies to all causes of failure to learn to read, not just to dyslexia. The majority of the 1 in 5 eighteen year olds who leave school unable to read as well as an average 11 year old, are not dyslexic, but they have been disadvantaged by a toxic combination of low general ability, poor teaching, lack of family support, truancy, and/or other social factors. The minority of these children who might be dyslexic cannot be distinguished from the socially disadvantaged on the basis of their phonological deficit alone [12].

On top of this, the classical criterion for DD of showing a discrepancy between reading and oral skills has been undermined by the claim that oral comprehension and non-verbal reading skills cannot be measured reliably. This has occurred despite a century of research into measures of general intelligence (‘g’) that have shown clearly that oral communication and non-verbal reasoning skills can indeed be accurately measured [13]. Nevertheless, dyslexia assessors have been advised that they should not try to use discrepancy criteria to diagnose dyslexia.

These factors—the takeover by psychologists, the emphasis on phonological awareness measures, and attacks on the discrepancy criterion—have left the diagnosis of developmental dyslexia in limbo, to such an extent that a sizeable number of educationalists have decided to abandon any attempt to diagnose it at all, because they have come to believe that DD does not exist. Yet, there is more and more research evidence that Hinshelwood and Morgan were correct. Developmental dyslexia does exist and a proper understanding of its causation should lead to better techniques for diagnosing and treating it.

## 3. New Theories

To overcome this confusion about how to diagnose dyslexia, a plethora of new theories about what causes it have been introduced. Any worthwhile theory should not only collect a summary of what we already know about the phenomenon, but it should also suggest its likely causes. The recent explosion of theories about DD is testament to how little we really know about the normal development of reading, let alone in developmental dyslexia. These new DD theories can be divided into 3 broad categories, psychological, signal processing, and neurological, but it is argued that only the latter can really provide useful pointers to likely causal brain mechanisms.

## 4. Phonological Theory of Dyslexia

The main psychological theory about dyslexia that has now assumed such dominance is the phonological theory. Its main weakness is that it is not really a theory at all in the sense of providing an explanation for the condition; it does not provide any hint as to its cause [12]. Indeed, the theory is almost a tautology, a circular argument, merely repeating using different words, that the children do not learn to read because they have not managed to acquire the phonological principle. However, every child who is failing to learn to read is failing to acquire the phonological principle. In order to help these children, we need to know in detail why each one of them fails to acquire it.

## 5. Rapid Automatised Naming (RAN) and the ‘Double Deficit’

In 1976, Martha Denckla discovered that people with true developmental dyslexia could be distinguished from poor reading from other causes, often called ‘garden variety poor readers’, by their slowness at ‘rapid automatised naming’ (RAN), i.e., being able to name out loud, as fast as possible, lists of letters, numbers, or pictures [14]. Maryanne Wolf then showed that this was not a phonological problem in the sense of learning the right phonemes, but that it was due to the slowed production of the correct sounds, i.e., it was a speech timing and fluency problem [15]. It then became clear that most dyslexics have this RAN deficit together with the phonological problems mentioned earlier. Thus, Maryanne introduced the ‘double deficit’ hypothesis [16]. Again, however, this is only descriptive and does not explain why dyslexics have these problems.

## 6. Reduced Visual Attention Span

Both Rudolph Berlin and Pringle Morgan thought that dyslexia was mainly a visual processing problem; thus, they often called it ‘word blindness’. However, since then, it has become clear that, in many dyslexic people, visual problems are not dominant. Nevertheless, it is now clear that reading starts with learning to sequence the letters in a word [10], and many children do have visual processing problems of various sorts [17,18]. Sylvianne Valdois advanced the hypothesis that dyslexics often may have a limited ‘visual attention span’ [19]. This is consistent with the increasing evidence showing that their deployment of visual attention is slow and less accurate [20], but again, by itself, this gives us no clue as what causes this deficit in visual attention, specifically in developmental dyslexia.

## 7. Visual Stress

Another suggestion is that many dyslexics suffer from ‘visual stress’, probably not as the main reason for their problems but often contributing to them [21]. Viewing high contrast, coarse (low spatial frequency), black and white stripes is quite uncomfortable for many people; the stripes can appear to move around, glare, or fade in some places and ‘strain’ the eyes, often causing eye and headaches. This is most likely because they overstimulate the system of visual neurones, the magnocellular timing system, which is responsible for rapidly signalling the moments when visual events occur. Some people are particularly sensitive to this kind of stimulus, and the black and white stripes formed by a page of text is precisely the kind of pattern that stimulates their visual systems maximally and may cause them discomfort. This appears to particularly be the case in some people with dyslexia. These uncomfortable symptoms can often be alleviated by viewing the page through coloured filters, most often deep yellow or deep blue, though there seems to be great individual variation, and there is much scepticism about whether these effects are purely placebo [22]. Again, however, clear as these symptoms are in some people, the visual stress theory offers no pointer to their causation.

## 8. Sensory Signal Processing

The next group of theories is concerned with signal processing aspects of reading. Any sensory system has the problem of isolating a signal of interest about what is going on outside the head from both ‘noise’ in the external environmental that can obscure the stimulus and also from internal noise, which is added to the signal at each stage by the brain’s own neural processing. Thus, a new theory about dyslexia is that each neural processing stage adds more noise than normal or is worse at separating the signal from the noise—the ‘noise exclusion hypothesis’ [23,24]. This is most easily demonstrated by the effects of distractors. Compared to typically developing readers, people with dyslexia have been found to be particularly poor at detecting stimuli in the presence of irrelevant distractors. Of course, the question then arises as to why they are so poor at ignoring the distractions. The answer, slow and inaccurate control of perceptual attention, brings us to the nature of attention, to which we will return.

Another theory involving signal processing limitations is that of ‘slowed Bayesian statistical learning’. This impedes the effective use of ‘priors’. Common experience of how a cause, a prior, is usually followed by the same effect, is routinely incorporated into our calculations to create an optimal program to achieve the skills required for reading and their successful automatization [25]. At present, there is not much evidence in favour of this hypothesis, and again it really only redescribes failure to acquire the necessary orthographic and phonological skills for reading; thus, it is not really a theory in the sense of suggesting the causes of these failures.

## 9. Sensory Temporal Processing

Another description of what is missing in a dyslexic person’s repertoire of reading skills is presented by the ‘temporal processing’ deficit theory. Nearly 40 years ago, Paula Tallal was the first to suggest that phonological weaknesses may actually be caused by an underlying auditory temporal processing deficit [26]. She revealed this by measuring in both the auditory and the visual modality the shortest gap that subjects could detect in an otherwise continuous stimulus (their gap detection threshold) or by judging the temporal order in which stimuli were presented. Such a temporal processing disorder in the visual or auditory domains, or both, has now been found in most people with developmental dyslexia [27]. Thus, it appears that impaired neural timing may be the actual root cause of developmental dyslexia itself, and so, we have to ask ourselves what the neural basis of this abnormality might be.

## 10. Neurological Theories

This brings us to the neurological theories attempting to explain the aetiology of developmental dyslexia. The 19th Century. originators of the concept clearly thought that it was due to failure to properly develop the specialised visual circuits underlying reading and spelling under genetic control. But we should not overemphasise the role of genetics. In both typically developing readers and dyslexics, their genes account for only half of the variation in their reading ability, and the environment they have experienced throughout childhood accounts for the remaining half. In other words, effective teaching at school and warm parental support, not just regarding reading but by providing a nurturing and encouraging home life, seem to be as important as heredity for typically developing readers and also for dyslexics. Nevertheless, all the evidence to date suggests that it is their genetic inheritance that sets the background to the failure of their reading connectome to develop properly [28].

## 11. The Reading ‘Connectome’

Successful development of the orthographic/phonological connectome seems to involve the repurposing of pre-existing connections, particularly in the left hemisphere, to mediate reading [29]. In people with dyslexia, however, their genetic inheritance seems to cause these new connections to fail to develop properly. Paulesu et al. described this, perhaps a little misleadingly, as a ‘disconnection’ syndrome [30]. This is misleading because it turned out that the problem is not the disconnection of previously established connections, but rather it is the failure to develop the correct specific connections for reading in the first place [31].

Most important developments in science have followed advances in methodology. The most significant recent technical advance in neuroscience has been the development of neuroimaging, in particular functional magnetic resonance imaging (fMRI), which has enabled regional transient increases in blood flow in the brain to be measured accurately in living subjects. Over a century ago, Roy and Sherrington showed that brain regions that are particularly active show increases in local blood flow [32], and modern advances in magnetic resonance techniques now allow us to follow these increases over time in courses of seconds in living subjects [33]. In addition, the introduction of ‘diffusion tensor analysis’ (DTI) has enabled the tracing of the ‘functional connectivity’ between regions, i.e., how the increased activity in one region is followed by increases elsewhere, presumably because of the activation of effective connections between them. Thus, the development of connections specifically mediating reading, the reading ‘connectome’, can now be followed in children by repeat imaging every few years [34]. These studies confirmed that Hinshelwood and Morgan were right. In developmental dyslexia, the specialised connections in the left hemisphere mediating reading fail to develop properly, although the precise nature of this disturbance may differ from individual to individual [35], and this applies particularly to differences between Chinese and alphabetic scripts [36].

## 12. The Cerebellum

Until recently, the magnets used for such imaging were only deep enough to image the cerebral cortex, but over the last few years, the brainstem and cerebellum have become included. In particular, these studies have demonstrated that the cerebellum plays a crucial role in the development of the reading connectome and of its operation in fluent readers [37]. Hence, as was first suggested by Fawcett and Nicolson [38], it seems that dyslexia is associated with reduced activity of the left cerebellum. Actually, it is probable that it is not the development of the cerebellum itself that is at fault, but rather the imprecision of the sensory timing inputs upon which its function depends [39].

The main function of the cerebellum is now believed to be to compute and maintain ‘internal models’ of movement in order to control the precise timing and force of the muscle actions required to achieve them skillfully and automatically [40]. These models depend on timing information supplied both directly by ascending projections from receptors in muscles and joints and from the visual, auditory, and somatosensory systems via the cerebral cortex. In humans, it is now clear that these internal models are not only used to control actual movements but also for thinking. ‘Thought is but movement confined to the Brain’, was Sherrington’s aphorism. For communication, speech, and reading, the internal models represented in the cerebellum may be used for cognition [41] to predict the consequences of potential movements without actually executing them in order to judge their likely outcome and, hence, suitability.

## 13. Temporal Processing Deficit

This brings us back to the temporal processing deficit theory of dyslexia. Our discussions of the many theories about the causes of developmental dyslexia all converge on the suggestion that the basic physiological mechanism that underlies it, and is therefore probably its root cause, is impaired temporal processing, but this term is too broad; it could cover anything from reading a clock to estimating a time interval. The studies that have contested the idea that DD has a temporal processing basis have often tested timing judgements [42], which are very different from the accurate signalling of the timing of events. We must therefore specify more precisely which timing functions are actually required for reading. Accurate signalling of the timing of when the eyes or visual attention alight on each letter in the written word is what is essential for determining the sequence of letters in the word [43]. Likewise, signalling the precise time at which each phoneme is heard enables the order of the sounds in the spoken word to be correctly sequenced [44]. Thus, the precise timing of these visual and auditory events is crucial for sequencing the letters and sounds in a word correctly, and imprecision of these magnocellular timing signals may underlie all the manifestations of dyslexia that we have considered.

This is not the place to discuss the wide variety of genetic, biochemical, anatomical, physiological, psychophysical, and behavioural evidence that justifies this conclusion, as it has been reviewed in detail several times recently [43,45,46,47]. Instead, here, we discuss two of the most common arguments questioning the timing deficit theory, namely its failure to explain the allegedly selective phonological deficit in DD, and the ‘chicken vs. egg’ problem—what is the direction of cause vs. effect?

The phonological theory of DD posits that it is due to a specific inability to grasp the phonological principle, specific in the sense that it is the development of the concept of phonemes itself that is affected and nothing to do with the quality of the sensory inputs that underlie its development. However, as noted earlier, everybody who fails to learn to read, whether dyslexic or not, has failed to grasp the phonological concept because only those who have grasped it can learn to read, i.e., it is inherent in learning to read. Thus, failure to do so cannot be used to distinguish dyslexics from those for whom social disadvantage has caused their difficulty with learning to read [12]. Furthermore, labelling dyslexics as having a phonological weakness is very likely to subject them to a rigorous teaching regime consisting of systematic phonics, which may not help them at all [48]. What we need to do is to discover what is causing each failure so that we can concentrate our efforts on what might help each of them individually, whether or not they are dyslexic.

## 14. Chicken or Egg?

The question of which is chicken and which is egg, i.e., the direction of cause vs. effect, permeates much of the discussion about causes of dyslexia [49] and whether dyslexia can be reliably distinguished from the other more numerous causes of reading failure. It is often argued that the sensory deficits that we have been discussing could be the consequence rather than the cause of dyslexic children’s failure to learn to read because they simply have not had so much reading experience, and thus, their sensory systems have not been ‘tuned up’ for reading [50].

## 15. Cohort Studies

Undoubtedly, learning to read must have an effect on the connections underlying it, which is the whole point of education, but there is now abundant evidence that the direction of cause and effect is mainly early congenital brain anomalies that cause sensory processing deficits, in turn causing reading difficulties much later. The most powerful way of determining the direction of causality in these circumstances is to use a ‘cohort study’, i.e., to follow a cohort of children who are at genetic risk of dyslexia from before they begin to learn to read to age 7 or 8. At this latter time, about half the children will be reading well, having not inherited vulnerability genes; the other half will be failing. Two detailed studies of this sort were undertaken in Holland and in Finland. They both showed that neurological differences manifest themselves in infants and, even in new born babies, in those who are going to go on to become dyslexic later on. 

In the Dutch study of children at family risk, habituation to a simple visual stimulus, which was neither alphabetic nor even letter-like, measured by visual evoked potentials at the age of 4 predicted whether the child would be identified as dyslexic by the age of 8 [51]. In other words, these children were clearly exhibiting visual processing abnormalities long before they began to learn to read, and these led to their later reading failure. This is strong evidence that their visual processing was the main cause of their reading problems, rather than vice versa.

In the case of the Finnish studies, when imaged in their mothers’ wombs even before birth, auditory processing anomalies were seen in the infants who later turned out to become dyslexic [52]. The magnitude of these abnormalities predicted the amount of the phonological deficit these children eventually experienced at age 8. Thus, this study provided yet more strong evidence that congenital brain abnormalities caused the later reading problems.

Both Dutch and Finnish are ‘transparent’ languages, i.e., their spelling translates into their sounds reliably. In English, on the other hand, 50% of common words are irregularly spelled, and thus, their spelling does not indicate how they should be pronounced. Instead, their visual form must be remembered. Therefore, too much emphasis on phonics at the expense of visual whole word recognition does not help many English dyslexics, but it could be argued that, whilst pre-reading brain differences have been clearly shown for Finnish and Dutch dyslexics, the same has not been shown for English or, as an example of an even more ‘irregular’ script, Chinese. Although detailed dyslexia cohort studies have not been completed for English or Chinese children as yet, already there is enough circumstantial evidence to be confident that, for these two scripts, sensory processing deficits in infancy precede and predict reading failure much later [49,53].

## 16. Intervention Studies

Another way of settling the direction of cause and effect for sensory performance is to see whether an intervention that improves it is followed by improved reading, with appropriate controls for placebo effects. Teri Lawton developed a technique based on the known properties of the M-system for training poor readers with an initially weak M-system [54]. She uses a low-contrast, low-spatial frequency, moving grating presented against a static background grating of higher contrast and spatial frequency. Subjects report the direction of motion whilst the program iteratively reduces the contrast of the moving grating as their threshold for correct responses improves. This training procedure greatly helps the children to improve their M-sensitivity. At the same time, she measures the children’s current motion sensitivity, how this improves with her training, and whether this enhances their reading progress. This procedure has shown clearly that this M-cell training helps the majority of dyslexic children improve their M-sensitivity and thereby make considerable progress with their reading [55]. These results provide convincing evidence that their improved M-function was what enabled their improved reading, rather than the other way around.

## 17. Is Developmental Dyslexia Multifactorial?

Taken together, these cohort and intervention studies have clearly established that impairments in temporal processing precede a child’s failing to learn to read. Therefore, they support the hypothesis that timing disorder contributes causally to the children’s failure. There are a number of studies at a psychological level, however, that suggest that reading failure is multifactorial and that there is unlikely to be a single mechanism underlying dyslexia. It is certainly true that some people with DD have more visual problems, while others have more auditory problems or a mixture of both, but as explained earlier, these differences probably derive from a basic temporal deficit that affects these two systems to different extents in different individuals for unknow reasons. Clearly, these need further research.

In general, studies that set up a psychological level statistical model fail to take into account the underlying neuroscience involved. To take a specific example, O’Brien and Yeatman [56] confirmed that a phonological deficit cannot explain all aspects of DD but went on to define three orthogonal visual, phonological, and decision-making clusters that are statistically partially independent. However, this approach ignores the well-evidenced dependence of phonological skills on visual letter sequencing [10], and the evidence that, in individuals, their visual timing deficits predict their auditory ones [57]. Analogous arguments may apply to other such models [58,59].

## 18. Parvocellular Proliferation

Most of the evidence considered so far supports the idea that the impaired development of magnocellular timing neurones in the brain is the ultimate cause of developmental dyslexia. Hence, this deficit underlies all the theories we considered above. The converse of this is that other neurones may be able to proliferate to fill the space vacated by the absent magnocells. More than 90% of the new neurones born in the foetal brain in the last few months of pregnancy fail to thrive and are eliminated jn early infancy because they fail to make successful and useful connections [60]. This means that the most numerous neurones, parvocells (parvus, Latin for small), may be able to flourish more than usual. Since parvocells are normally more extensively connected than magnocells, their superabundance in dyslexia implies that their connectivity might be even greater. Thus, the magnocellular timing deficit could be matched by a superabundance of parvocells in DD.

Although there is not so much evidence in favour of this proposition because most studies look for deficits in dyslexic brains, not strengths, there are a number of studies that have shown clearly that people with dyslexia have superior performance on tests that specifically assess parvocellular functions. For example, in the same studies in which Lovegrove showed dyslexics to have lower magnocellular sensitivity than ordinary readers to high temporal but low spatial frequency modulated gratings, he showed that they actually had a much higher sensitivity to parvocellular stimuli (high-spatial but low-temporal frequency gratings) [61]. Thus, whilst dyslexics have reduced steady state visual evoked potentials at magnocellular frequencies, they have enhanced responses at parvocellular frequencies [62]. Others have shown dyslexics to have better red and green colour discrimination, particularly in the peripheral visual field [63]. Likewise, they have shown to have better detail vision in the periphery of the visual field [64]. Thus, there is clear evidence that dyslexics may develop larger numbers of parvocells particularly in the visual field outside of the fovea.

## 19. Positive Dyslexia

There is also a large, mainly anecdotal, literature suggesting that people with dyslexia are overrepresented compared to ordinary brains in a specific set of professions, namely aesthetic and artistic, entrepreneurial businesses, practical engineering, and mathematics and computing. There is a long list of famous and exceptionally talented probable dyslexics, ranging from Caesar Augustus [65], via Leonardo da Vinci [66], to Einstein, Winston Churchill, and Stephen Spielberg. On the face of it, there is nothing obvious to link their varied occupations together. One suggestion is that dyslexics take refuge in professions that do not require much reading, but nowadays, every profession carries a large load of literature. Actually, if dyslexics avoid it as much as possible, this could not explain why they are often so successful in those professions. In fact, this argument leads to the possible conclusion that dyslexics may actually be inherently more talented and creative in these pursuits.

## 20. Creativity

Clearly, the next question must be: might these creative talents derive from dyslexics’ superabundance of parvocells? It is often suggested that people with dyslexia are constitutionally more creative than others, but the most widely used assessment of creativity, the Torrance test, was not developed with the properties of parvocells in mind. Hence, most studies have found that dyslexics are more creative in some aspects of life but not in others. Thus, overall, these studies have failed to demonstrate that dyslexics are more creative than ordinary people.

## 21. Impossible Figures

However, there are a few tasks in which dyslexics have been reliably found to be faster and more accurate than non-dyslexics. ‘Impossible’ figures are drawings of objects that cannot exist in reality, such as the impossible stairs and waterfalls depicted by M.C. Escher or Schuster’s impossible trident. When asked to decide whether drawings of objects were physically possible or impossible, Von Károlyi found that dyslexics were much faster and more accurate in this task [67]. Male dyslexics have also been shown to be particularly good at identifying shapes in ambiguous figures, remembering and reproducing designs and complex figures, and at recreating and navigating in a virtual environment [68].

The next obvious question therefore is whether the tasks in which dyslexics do show clear superiority are ones that particularly call on parvocellular function. As we have seen, dyslexics’ P-cell advantage probably gives them a wider and better detail vision of pattern and colour, particularly in the periphery. Thus, they can often pick up more detail over a wider area in an instant than ordinary brains can. This is often called ‘holistic’ vision because its possessors can see a larger scene in detail at a glance. It is also sometimes called ‘helicopter’ vision, which means to be able to imagine a whole scene from above in all its elements and all its detail and, hence, to be able to discern what ordinary, more tunnel-vision, brains might fail to see. Even more importantly, this can enable many dyslexics to spot anomalies in a pattern that may indicate weakness or error. Given this holistic skill, it is not surprising that people with dyslexia can see all Escher’s waterfalls at once and quickly decide that they could not work together. Likewise, they may be able to see both ends of Schuster’s trident simultaneously and to quickly determine that it has three elements at one end, but only two at the other end, and thus is physically impossible to exist [67]. Likewise, successfully discerning shapes in indistinct pictures would be greatly assisted by the holistic vision presentation of the whole drawing simultaneously [68].

## 22. Auditory ‘Parvocellular’ Perception

Both auditory and visual timing systems are affected in dyslexia; hence, parvocells may proliferate in both. What might the perceptual advantage be that is imparted by dyslexics possessing increased numbers of auditory parvocells that would be analogous to their gaining holistic vision? As for the visual cortex, dual magnocellular and parvocellular streams pass forward in the brain from the primary auditory cortex [69]. The auditory magnocellular timing stream in dyslexics is compromised, which is why they tend to be poor at picking up rhythm [70], but they are better, for example, at detecting the combination of components in a musical chord [71]. This holistic perception of music seems to bestow on them a better appreciation of the coloration, timbre, and emotional tone of music, and it enables some to take in the whole structure of a tune or harmony all at once. Thus, even though they usually have great trouble sight reading, they can often remember a whole tune immediately and reproduce it correctly on the piano or guitar. Thus, whilst dyslexia puts obstacles in the way of learning to read music, it nevertheless assists some to become celebrated musicians.

## 23. Abstract Cognitive Style

The observations that follow are difficult to test scientifically because the skills involved are so rare that tests for them have not been developed. It would be best to treat them as speculations at present. Metacognition is ‘thinking about how you think’. It seems that parvocellular proliferation in dyslexics may confer on them a distinctive metacognitive style that is holistic in an abstract sense. Many seem to be able to take into account all the elements of a problem or situation together and thereby see clearly which fit together into a recognisable pattern and which do not. For example, dyslexics are notoriously good at judging personality, taking all factors into account simultaneously, not just one at a time, and coming to accurate conclusions. This cognitive style fosters ‘lateral thinking’, originality, creativity, inventiveness, and seeing every element of a problem all at once. Einstein was famous for his even more abstract mind set; he is said to have been able to see patterns in 10 or 12 dimensions, imagine their outcomes, and thus predict their unexpected properties.

## 24. Conclusions

More than a century ago, the concept of developmental dyslexia was introduced, but it is now under sustained attack because the now-dominant phonological theory of its causation fails to distinguish DD from the many other possible causes of failure to learn to read. However, our consideration of the many and varied theories of its causation that have recently been introduced converges on the simple idea that underlying all of them is imprecise signalling by magnocellular neurones of the timing of the visual and auditory events involved in reading. This timing deficit can distinguish dyslexia from other causes of reading failure that do not show such timing problems. The converse of this magnocellular deficit in dyslexia may be parvocellular proliferation in them, which has also been clearly demonstrated. This compensation may explain ‘positive’ dyslexia, which is the exceptional creative, artistic, entrepreneurial, communication, and engineering talents that some dyslexics display, and it emphasises that dyslexic talents should be valued, prioritised, and nurtured. The unsustainability of the modern world needs their imagination, innovation, and creativity to help us survive [72].

## Data Availability

Not applicable.
